# Assessing the effectiveness and observing fidelity of a psychosocial support program for Rohingya refugee mothers and their children in Cox’s Bazar, Bangladesh

**DOI:** 10.1192/j.eurpsy.2023.776

**Published:** 2023-07-19

**Authors:** K. Le Roch, A. J. Nguyen, K. S. Rahaman, M. Lasater, S. Barua, C. Lee, M. Schojan, L. Clouin, S. M. Murray

**Affiliations:** 1 Mental Health and Psychosocial Support, Action contre la Faim, Paris, France; 2 School of Education and Human Development, University of Virginia, Charlottesville, United States; 3 Mental Health and Psychosocial Support, Action Against Hunger, Cox’s Bazar, Bangladesh; 4 Department of International Health; 5 Department of Mental Health, Johns Hopkins Bloomberg School of Public Health, Baltimore, United States

## Abstract

**Introduction:**

Despite the well-recognized risk poor maternal mental health poses to early child development, it is still rarely addressed in global health programming, especially in humanitarian settings where access to health and mental health infrastructures may be limited. Recognizing the critical role of maternal psychosocial wellness in addressing the health and development of children in conflict, Action contre La Faim/Action Against Hunger (ACF) developed the Baby Friendly Spaces (BFS) program. BFS is a holistic, evidenced-based psychosocial support program that aims to enhance mothers’ wellbeing, internal resources, and child caring skills in order to create a buffer against the deleterious health and developmental impacts of conflict on children.

**Objectives:**

In Bangladesh, we sought to evaluate the effectiveness of a psychosocial support program for Rohingya refugee mothers and their malnourished children under two years old living in Cox’s Bazar’s camps.

**Methods:**

For this study, we used a matched pair randomization, where ten BFS program sites were allocated to either continue providing services “as usual” or to an “enhanced BFS program” after re-training and providing continuous supportive supervision of the BFS staff throughout the trial period. 600 mothers and their children were enrolled in the study and attended psychosocial stimulation activities related to child care practices and care for women. Data were collected at baseline and 8-week follow-up. Primary outcomes included maternal distress and wellbeing, functioning, and coping. For implementation purpose, a survey was administered on confidence at work for all BFS staff and a fidelity observation assessment was conducted.

**Results:**

Relative to “as usual” sites, mothers in enhanced implementation sites reported greater reductions in distress (B=-.30) and improvement in wellbeing (B=.58). These differences were small, but marginally significant (p=.058; p=.038) with standard estimation; There was no significant difference between the two groups for daily functioning and coping. BFS providers in “enhanced BFS program” reported higher confidence in service delivery than their colleagues (p=.01). Fidelity varied widely across different components, with some very high and some very low adherence. There tended to be better adherence to procedures in group versus individual sessions and for some specific activities across domains, for enhanced versus standard BFS.

**Conclusions:**

Findings highlight the value of innovative study approaches for real-world evidence generation. Small but feasible adjustments to implementation can both improve program delivery for maximizing impact. Consequently, low-intensity psychosocial support activities holds potential for reducing distress and improving subjective well-being of conflict affected mothers.

**Disclosure of Interest:**

None Declared

